# A feasible and reliable self-administered parental assessment of children’s lifestyle (SAPLACL): an ancillary study based on the VIF program

**DOI:** 10.1186/s13104-022-06069-1

**Published:** 2022-05-15

**Authors:** Jérémy Vanhelst, Valérie Deken, Gaëlle Boulic, Alain Duhamel, Monique Romon

**Affiliations:** 1 Univ. Lille, Inserm, CHU Lille, U1286 - INFINITE - Institute for Translational Research in Inflammation, and CIC 1403 - Clinical Investigation Center, 59000 Lille, France; 2 CHU Lille, ULR 2694 – METRICS: Evaluation des technologies de santé et des pratiques médicales, Univ. Lille, 59000 Lille, France; 3 Association FLVS, 59350 Saint-André, France

## Abstract

**Objectives:**

In children, achieving an acceptable degree of accuracy from dietary or physical activity (PA) assessments remains a challenge. Children tend to overestimate their time spent in daily PA and underestimate their dietary intake of fat and sugar. Because parents play a key role in family lifestyle decisions, including children’s food choices and PA levels, it is important to investigate the responses of parents regarding their children’s lifestyle habits. We aimed to develop a Self-Administered Parental Assessment of Children’s Lifestyle (SAPLACL) questionnaire and test its feasibility and reliability in 191 parents (29 fathers and 162 mothers).

**Results:**

For each part of the questionnaire, the rate of missing or improper responses ranged from 0 to 24%. The highest proportion of problems in understanding was reported for the dietary intake dimension, especially for snacking in front of the TV. Some difficulty was also found regarding the question on leisure PA. Test–retest agreement was observed in 54.7–100% of the respondents. Overall, the kappa coefficients were favorable. Thus, the parent self-report questionnaire is a valid and accurate tool for analyzing children’s lifestyle habits in France.

## Introduction

Obesity in youth is considered the main childhood health problem in Europe [1, 2]. In 2010, it was estimated that about 25% of European children (aged 6–9 years) were overweight or obese [1]. Many studies showed that the prevalence of overweight and obesity in French children and adolescents was stable [3, 4]. However, while the prevalence of overweight decreased, it still remained high in French youth [3, 4]. Overall, French monitoring/surveillance studies reported prevalence rates of overweight and obesity in youth that were similar to those found in European countries (20–25%). Obesity is related to numerous health problems that tend to progress from childhood to adulthood with considerable long-term health and economic burdens [5]. Moreover, obesity is caused by complex interactions between biological, developmental, behavioral, genetic, and environmental factors [6].

The benefits of healthy lifestyle habits are well documented [7]. Physical inactivity, sedentary behaviors, and dietary habits are widely recognized as the key determinants of health from childhood to adulthood [8–11]. Obesity is caused by the combination of a less active lifestyle, including sedentary behaviors, and a failure to reduce energy intake to match the reduced total energy expenditure arising from reduced physical activity (PA) [12]. Sleep habits may also have an impact on weight status. Studies have shown that short sleep duration and poor sleep quality are considered potentially modifiable lifestyle risk factors that may contribute to the development of obesity. Considering the numerous health consequences of the physical, social, and mental parameters in childhood obesity, there is a need to plan intervention and promotion programs around unhealthy lifestyle habits to reduce the prevalence of childhood obesity. Evaluation of these programs will enable an accurate assessment of these lifestyle habits. In children, achieving an acceptable degree of accuracy from dietary or PA assessments remains a challenge [13, 14]. Children tend to overestimate their time spent in daily PA and underestimate their dietary intake of fat and sugar [14, 15]. Because parents play a key role in family lifestyle decisions, including children’s food choices and PA levels, it is important to investigate the responses of parents regarding their children’s lifestyle habits [16–18].

Thus, this study aimed to develop a Self-Administered Parental Assessment of Children’s Lifestyle (SAPLACL) questionnaire and test its feasibility and reliability.

## Main text

### Methods

#### Study design and participants

This ancillary study used data from the *Vivons en Forme* (VIF; “live healthy”) program [19]. The VIF program is a community-based prevention program aimed at promoting healthier lifestyles among children and their families, and it involves the municipal services in charge of children’s education and care [20].

An informative letter was provided to parents explaining the objectives of the study and guaranteeing that the data would remain strictly anonymous and confidential. The reliability study was conducted over a 2-week period, requiring two participant contacts. During the first visit, the selected version of the questionnaire was completed, and demographic data were obtained. The participants completed the same selected version of the questionnaire up to 2 weeks later. Both questionnaires were completed by 65 parents (i.e., 130 questionnaires).

This study did not involve any intervention and was conducted on a volunteer basis. Data were retrospectively collected by the study organizational structure [19]. In this context, written informed consent was not required according to the French human research regulations [19]. All answers provided by the participants were anonymized and confidential.

#### Questionnaire

The questionnaire was prepared by a group of highly skilled professionals with experience in public health studies in the field of nutrition. A pretest was conducted to evaluate the clarity, comprehensiveness, and acceptability of each question, as well as questionnaire length and the quality and response rate for each question in an independent sample of 191 parents (father or mother). Questions were deleted or modified if they were completed by less than 80% of the participants. After each step, appropriate changes were made to produce the final questionnaire.

The final questionnaire consisted of 22 questions divided into the following three parts: part one—dietary habits of their children; part two—lifestyle habits of their children; and part three—PA and dietary habits of their children. An introductory part of this questionnaire included questions to elicit the demographic and social information, including date of completion, gender of the participant and their child, name of the school attended by and class level of their child, and city of residence.

The dietary habits of their children (part one, 11 questions) were assessed using dietary history (closed-ended questions). This part of the questionnaire contained questions about drinking habits and consumption of fruit, vegetables, dairy products, sweets, salted snack foods, and different types of beverages. Questions about the lifestyle habits of their children (part two, five questions) were divided into the following sections: (*i*) sleep habits, (*ii*) duration and mode of commuting to school, and (*iii*) sedentary behaviors. The answers were a simple choice according to several defined answers. Questions about their PA and dietary habits (part three, six questions) were divided into the following three sections: (*i*) sports practice, (*ii*) dietary intake during sedentary behaviors, and (*iii*) time and importance of PA and food preparation. A Likert scale and closed-ended questions were used for this part of the questionnaire.

At the end of this questionnaire was a section asking for information to build a profile of the parents. Information on the educational level of the mother and father was requested and then classified into one of three categories using a specific questionnaire adapted from the International Standard Classification of Education (ISCED, 2011). Educational level was scored as follows: 1, primary and lower education (levels 0, 1, and 2 in the ISCED classification); 2, higher secondary education (levels 3 and 4 in the ISCED classification); and 3, tertiary education (levels 5 to 8 in the ISCED classification). Then, the marital situation of the participant and the number of children in the household were also assessed.

### Statistical analysis

Categorical variables were expressed as percentages. The test–retest reliability was evaluated using the intraclass correlation coefficient for quantitative items, simple Cohen’s kappa coefficient for nominal variables, and weighted Cohen’s kappa coefficient for ordinal variables for categorical items. The values of intraclass correlation coefficient and kappa values are as follows: poor agreement of values, < 0.45; average-to-good agreement of values, 0.45–0.75; and excellent agreement of values, > 0.75 [21]. Statistical testing was performed at the two-tailed α-level of 0.05. Data were analyzed using SAS software version 9.4 (SAS Institute, Cary, NC, USA).

### Results

Sample characteristics of participants for assessing the reliability of the SAPLACL questionnaire are shown in Table 1. In total, 65 participants (10 fathers and 55 mothers) from three cities were included in this study.

**Table 1:** Sample demographic characteristics

	n	%
*City*		
Total	65	100
Roncq (Nord)	33	51
Saint-Martin-de-Crau (Bouches du Rhône)	11	17
Vieux-Condé (Nord)	21	32
*Gender of respondents*		
Father	10	15
Mother	55	85

#### Feasibility of the SAPLACL

From 216 eligible parents, 191 completed questionnaires were obtained, providing an overall participation rate of 88.4%. Mean response rates according to each part of the questionnaire (dietary, lifestyle, and PA habits) are described in Table 2. Missing or inappropriate responses for different parts of the questionnaire ranged from 0 to 24% (Table 2). Due to a poor response rate, some questions about the composition of snack foods consumed and the physical leisure activities pursued were reworded to improve the understanding.

**Table 2:** Mean rate response according to each part of the questionnaire

	Rate response (*%*)
Part 1: Dietary habits	
Take Breakfast during school day	87.7
Take Breakfast during school free day	87.7
What	98.5
Morning snacking	98.5
Lunch school	100
Afternoon snacking	98.5
Snacking front of the TV	95.4
Foods intake	87.7 to 98.5
Drink intake	76.9 to 95.4
Part 2: Lifestyle habits	
Sleep Week	87.7
Sleep Weekend	86.1
Mode of commuting to school	89.2
Screen	92.3 to 96.9
Part 3: Physical activity habits	
Physical Activity in Sport Club	76.9 to 90.8
Leisure Physical Activity	93.8
Awareness Physical Activity perception	90.8 to 95.0

#### Reliability of the SAPLACL

Test–retest reliability data for the questionnaire are presented in Table 3. Test–retest agreement was observed in 54.7–100% of the respondents. This agreement was generally confirmed using the kappa coefficients (Table 3), which were the following: 0.53–1.00 for dietary habits, 0.84–0.93 for lifestyle habits, and 0.66–0.87 for PA habits.

**Table 3:** Test–retest reliability of the questionnaire (retest after 2 weeks)

	Number of items	Agreement (*%*)	Reliability index^a^
Part 1: Dietary habits			
Take Breakfast during school day	1	100	0.81 (0.57–1.00)
Take Breakfast during school free day	1	100	1.00 (1.00–1.00)
What	9	67.2 to 90.60	0.65 (0.27–0.81)
Take Morning snacking	1	87.5	0.85 (0.75–0.95)
Lunch school	1	96.9	0.96 (0.91–1.00)
Afternoon snacking	1	95.3	0.55 (0.09–1.00)
Snacking front of the TV	1	96.8	0.86 (0.66–1.00)
Foods intake	9	54.7 to 81.2	0.53 (0.27–0.60)
Drink intake	6	56.1 to 95.2	0.63 (0.33—0.76)
Part 2: Lifestyle habits			
Sleep week	1	NA	0.84 (0.75–0.90)^b^
Sleep weekend	1	NA	0.84 (0.75–0.90)^b^
Mode of commuting to school	1	96.5	0.90 (0.76–1.00)
Screen	5	91.7 to 100	0.93 (0.82–1.00)
Part 3: Physical activity habits			
Physical Activity in Sport Club	1	94.9	0.87 (0.72–1)
Leisure Physical Activity	1	90.2	0.80 (0.65–0.95)
Awareness Physical Activity perception	1	NA	0.66 (0.50–0.78)

NA: not applicable,

^a^Reliability index indicates the Kappa value (95%Confidence interval (CI)) or the median (range) of Kappa individual items values in case of multiple items per dimension unless otherwise as indicated

^b^Intraclass correlation coefficient (95%CI)

## Discussion

This study aimed to develop a parent self-report questionnaire for exploring lifestyle habits in children and test its feasibility and reliability. The present study findings indicated that the parent self-report questionnaire can be considered acceptable and reliable in the assessment of children’s lifestyle habits.

Evaluating lifestyle habits in children is a complex challenge [22]. Many objective assessment techniques exist, but they tend to be difficult to set up for large cohort studies. Therefore, in some cases, questionnaires can be used because they are low cost, easily and rapidly administered, and enable large numbers of people to be tested simultaneously [22, 23]. Because the family is fundamental to assessing children’s habits, we decided to develop a parent self-report tool to assess children’s lifestyle habits, including dietary and lifestyle habits (PA, sedentary behaviors, and sleep). The major problem with questionnaires is that they are self-reported and subjective, which makes them susceptible to errors from aspects such as social desirability, lack of awareness, and perceptual bias. This can lead to over- or underreporting, particularly in youth [14]. Furthermore, parents do not always accurately report children’s habits [24, 25]. Our feasibility study showed that the parent self-report questionnaire was generally easy to complete. Dietary intake was the dimension where parents had more difficulty than other dimensions in understanding the question and providing a correct answer. Moreover, it was not surprising to have some problems of comprehension regarding the dietary intake topic. Indeed, some previous studies also reported difficulties faced by parents in accurately reporting the dietary habits of their children [24, 26]. An accurate assessment of dietary habits by parents is crucial to investigating the relationship between dietary intake and childhood obesity, which is a major public health concern. Therefore, some questions about snacking in front of the TV and the type of snacks consumed were reworded to improve the parents’ understanding. Regarding lifestyle habits, particularly those concerning sedentary behaviors, two items were added to the parent self-report questionnaire after the first stage of its construction—smartphone and digital tablet were added to the question on the time spent in front of a TV screen. Indeed, as demonstrated in two previous studies, the exposure and use of mobile media devices by children have dramatically increased [27, 28]. No other changes were made to the rest of the questionnaire. Findings regarding the small proportion of missing and inappropriate responses confirmed the feasibility of our parent self-report questionnaire to assess the PA, sedentary behaviors, and dietary intake of the children. Moreover, it is possible to easily assess children’s lifestyle behaviors in a large French cohort using this questionnaire, thus making the present study findings interesting.

Reliability is a key indicator of the quality of the assessment tool. This is especially important for self-report questionnaires because most epidemiological studies on lifestyle habits require the use of a questionnaire instrument to simultaneously assess a large number of participants. Therefore, reliability is an important issue in the choice of questionnaire instruments. The questionnaire used in the present study showed fair-to-excellent levels of test–retest reliability according to the established standards for reliability coefficients [29]. This good overall reliability allowed us to use the parent self-report questionnaire to assess children’s lifestyle habits in a large cohort during the same period. Our findings are in agreement with those of previous studies assessing children’s lifestyle behaviors (PA, sedentary behaviors, and dietary intake) using a self-administered questionnaire [30–32]. Moreover, a meta-analysis reported a moderate-to-strong criterion validity of sleep time questionnaires [33]. However, the reliability assessment of the questionnaires showed strong validation performance. The high reliability index found in the present study regarding the sleep time during weekdays and weekends confirms these findings reported by Nascimento-Ferreira et al. [33]. Therefore, the SAPLACL represents a good alternative to objective methods, most constraining, such as accelerometry, for assessing sleep habits.

Overall, the questionnaire used in the present study is an acceptable and reliable tool that allows the assessment of children’s lifestyle habits from the parents’ perspectives. These initial findings are promising and suggest that this instrument is suitable for use in identifying and prioritizing areas that require the implementation of health promotion programs aimed at improving the health of children.

## Limitations

The main limitation was the sample included in our ancillary study. The included samples were not necessarily representative of the French population as a whole. Indeed, the study did not use a randomized sample design. Regulatory and ethical constraints present a second major study limitation. It was not possible to record several additional clinical data on parents, such as body weight or body mass index, even though these parameters could have influenced the answers that they provided about their children’s lifestyle. Social desirability and social approval might have also biased our findings [34]. Both test and retest questions might also have been consistently answered based on an incorrect self-perception. Lastly, the study design did not include several characteristics of the study sample, such as age, sex, and weight status, which could impact our findings. The main strength of this study is the development of a short parent self-report questionnaire to assess children’s lifestyles. However, the present study findings should be treated with caution because of the lack of an external evaluation.

## Data Availability

The datasets used and/or analyzed during the current study are available from the corresponding author on reasonable request.

## Abbreviation

PA: Physical activity
